# Supplementary material for: The centrosomal protein 83 (CEP83) regulates human pluripotent stem cell differentiation toward the kidney lineage
Source: eLife. 2022 Oct 12;11:e80165. doi: 10.7554/eLife.80165 (PMC9629839; doi:10.7554/eLife.80165)

Fig. 1B: Gel of PCR for CEP83 detection between WT hiPSCs versus Transfected hiPSCs with gRNAs.

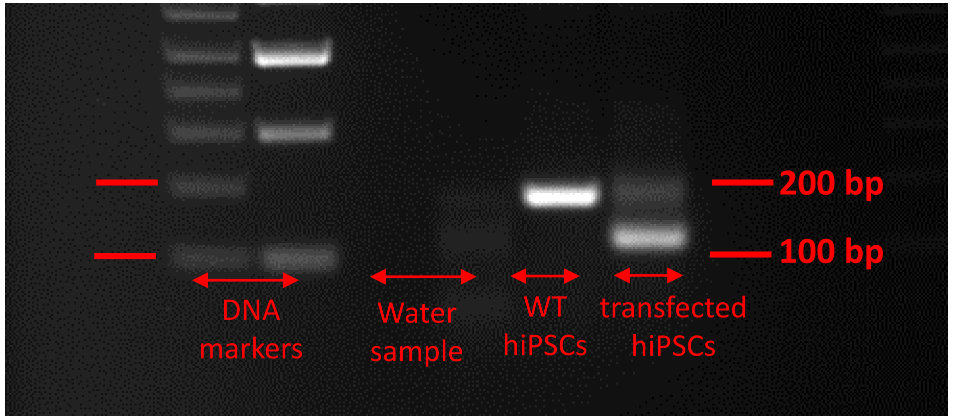

Fig. 1C: Gel of PCR for CEP83 expression in the selected *WT* and *KO* clones.

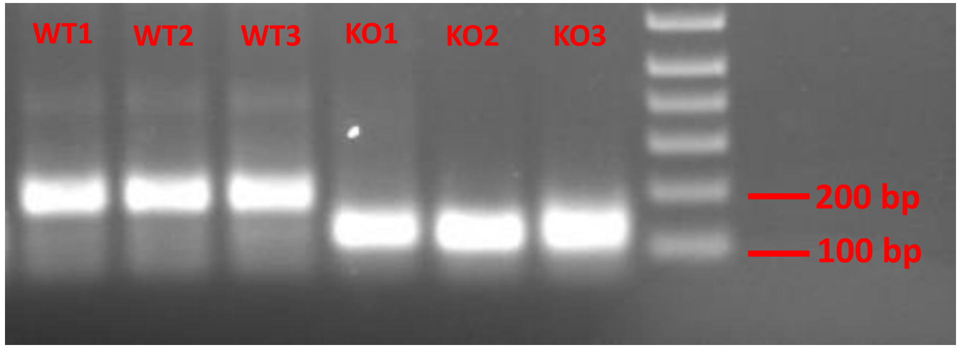

Fig. 1E: Western blot membrane for CEP83 protein detection in the selected *WT* and *KO* clones.

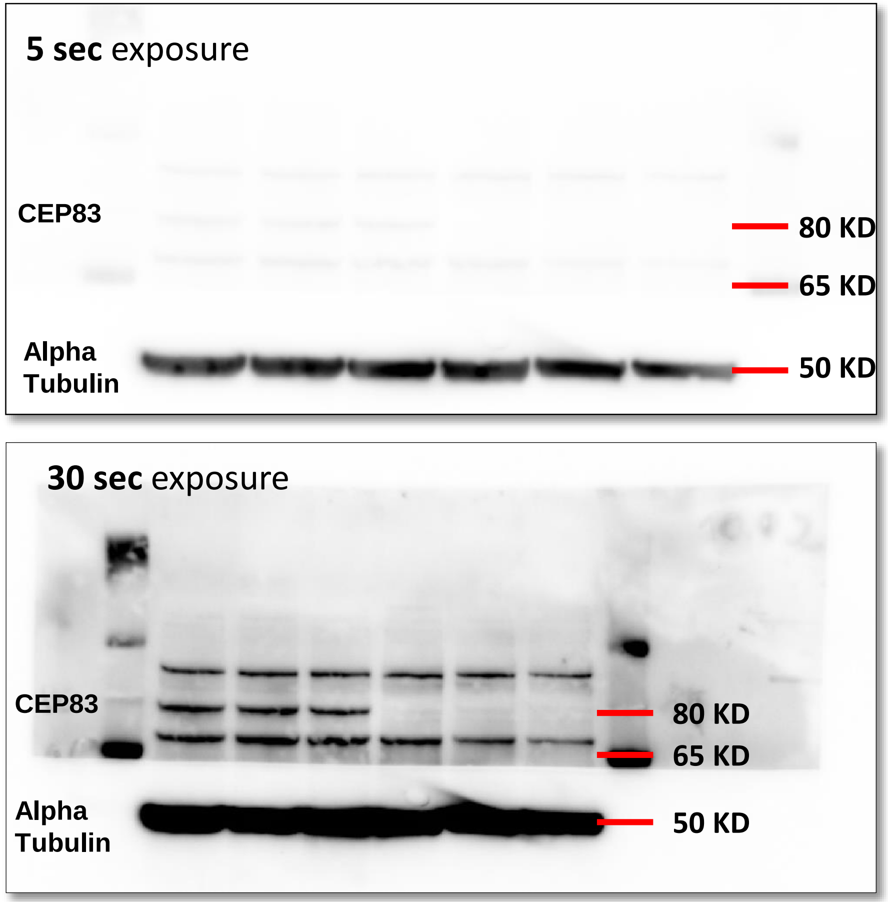

Supplement: Figure 1—source data 1. [file elife-80165-fig1-data1.pdf]
