# Supplementary figures and images for: The centrosomal protein 83 (CEP83) regulates human pluripotent stem cell differentiation toward the kidney lineage
Source: eLife. 2022 Oct 12;11:e80165. doi: 10.7554/eLife.80165 (PMC9629839; doi:10.7554/eLife.80165)

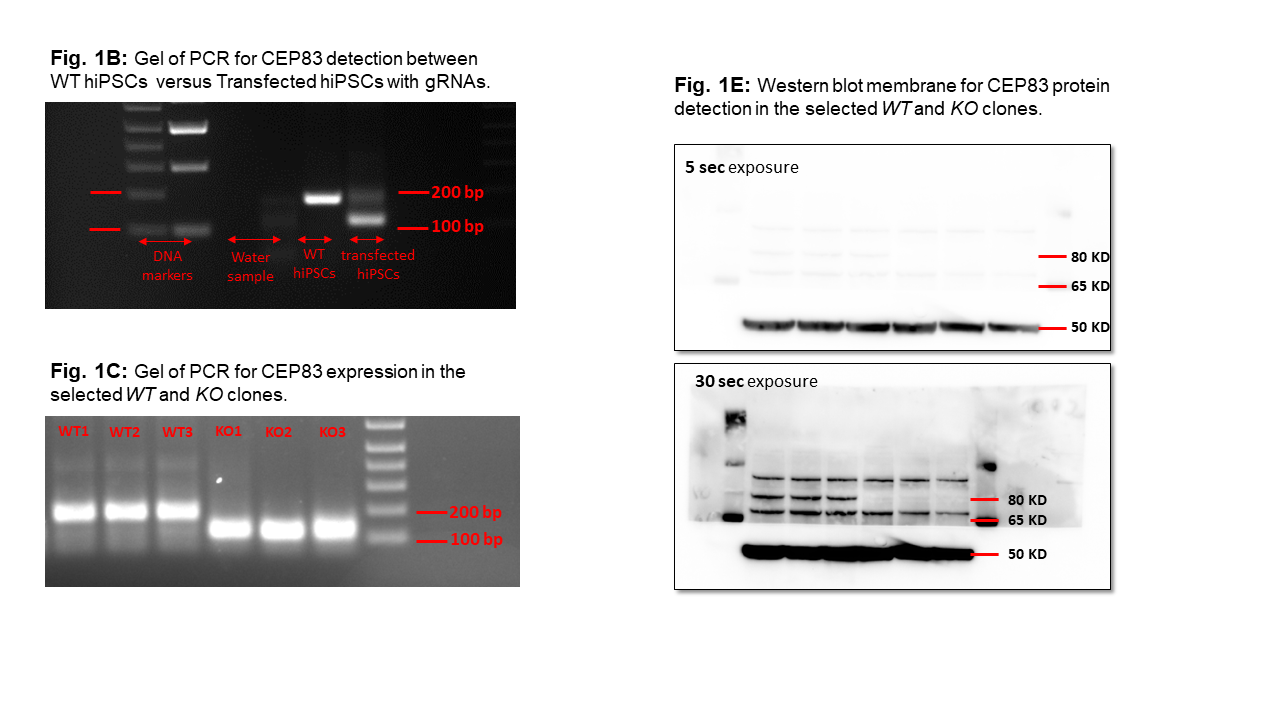

Supplement: Figure 1—source data 3. [file elife-80165-fig1-data3.zip › Figure1_B_C_D_source data/Figure 1- source data 1.png]

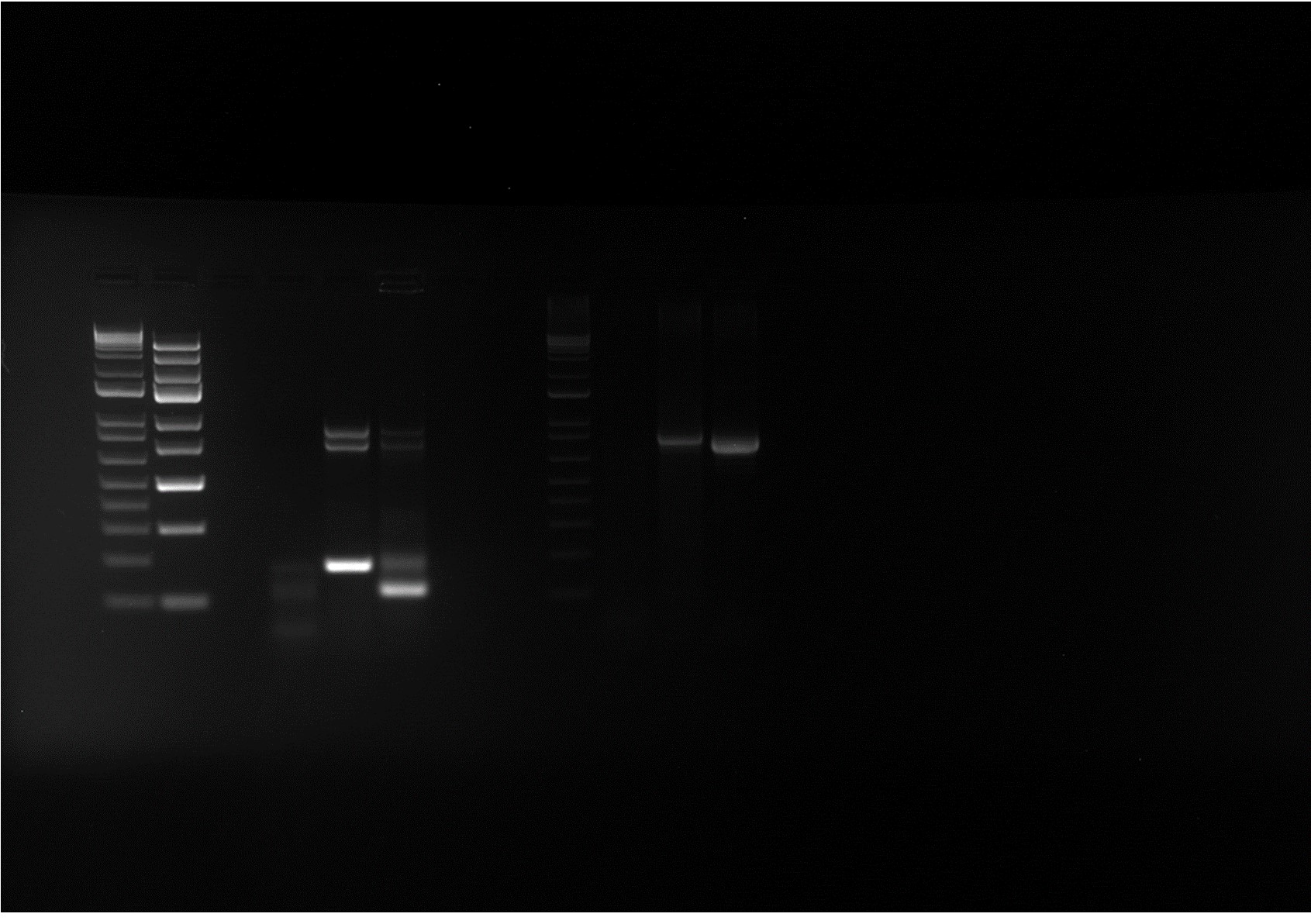

Supplement: Figure 1—source data 3. [file elife-80165-fig1-data3.zip › Figure1_B_C_D_source data/Figure_1B_raw_gel.jpg]

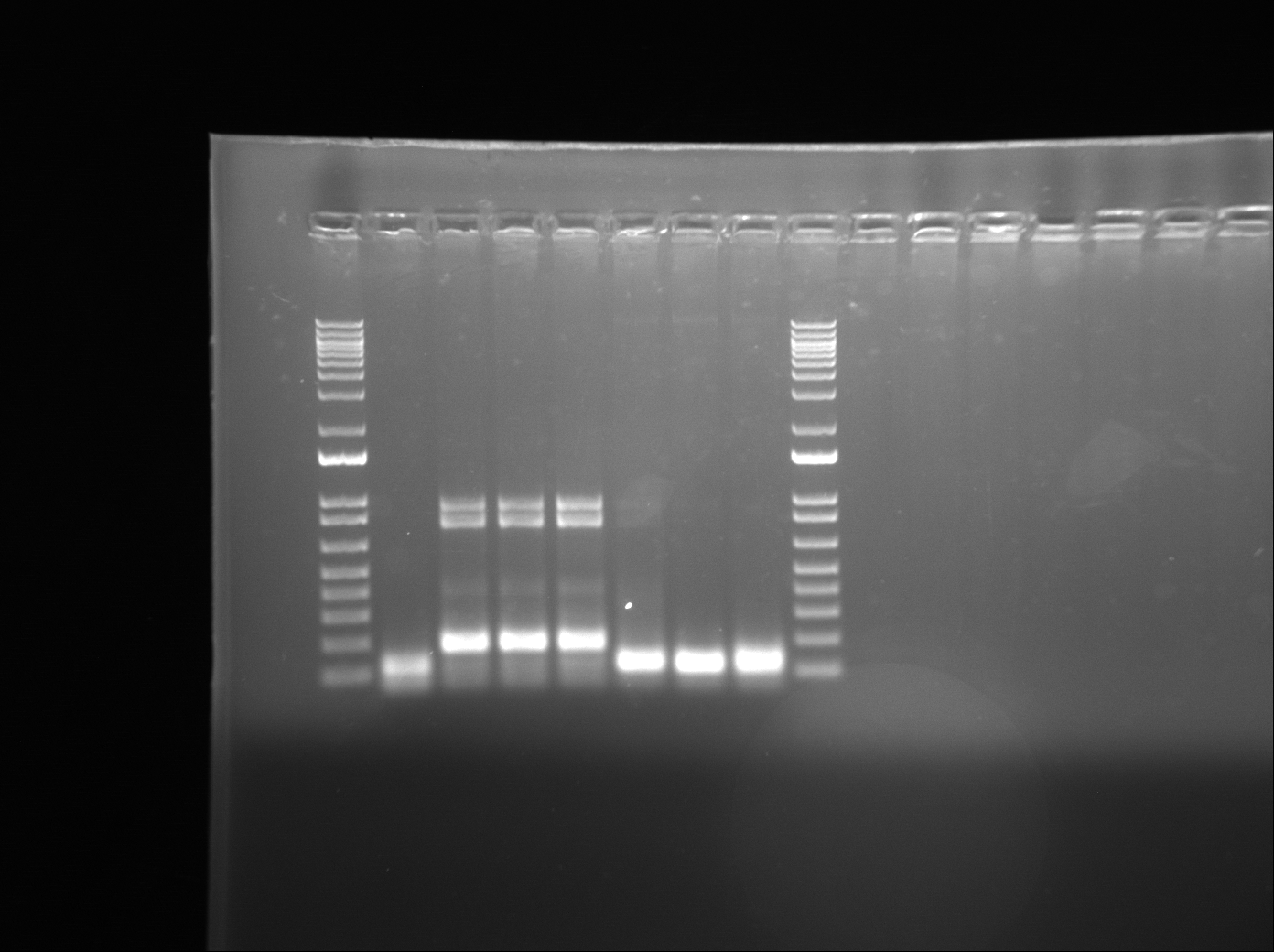

Supplement: Figure 1—source data 3. [file elife-80165-fig1-data3.zip › Figure1_B_C_D_source data/Figure_1C_raw_gel.TIF]

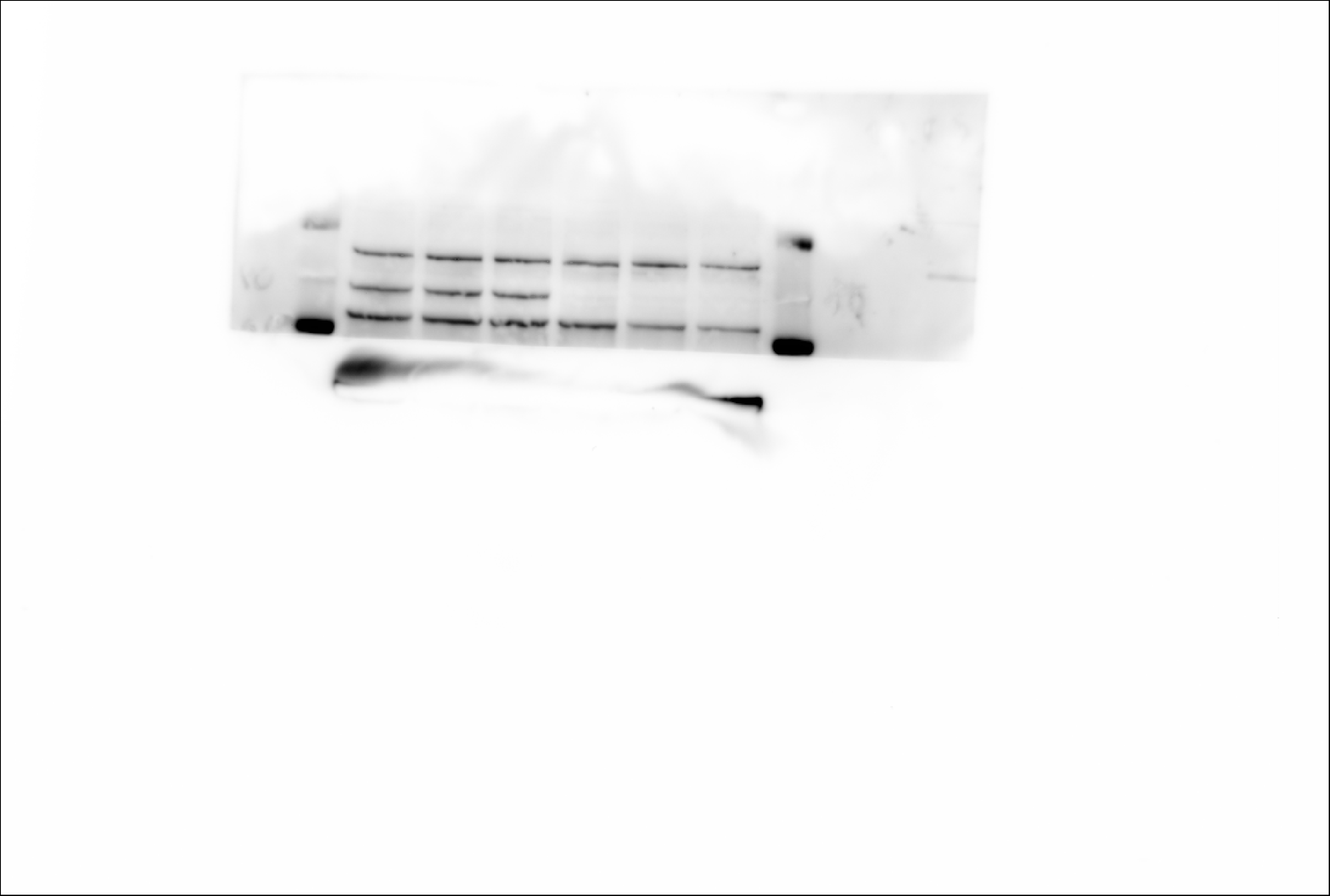

Supplement: Figure 1—source data 3. [file elife-80165-fig1-data3.zip › Figure1_B_C_D_source data/Figure_1D_raw_memberane1.tif]

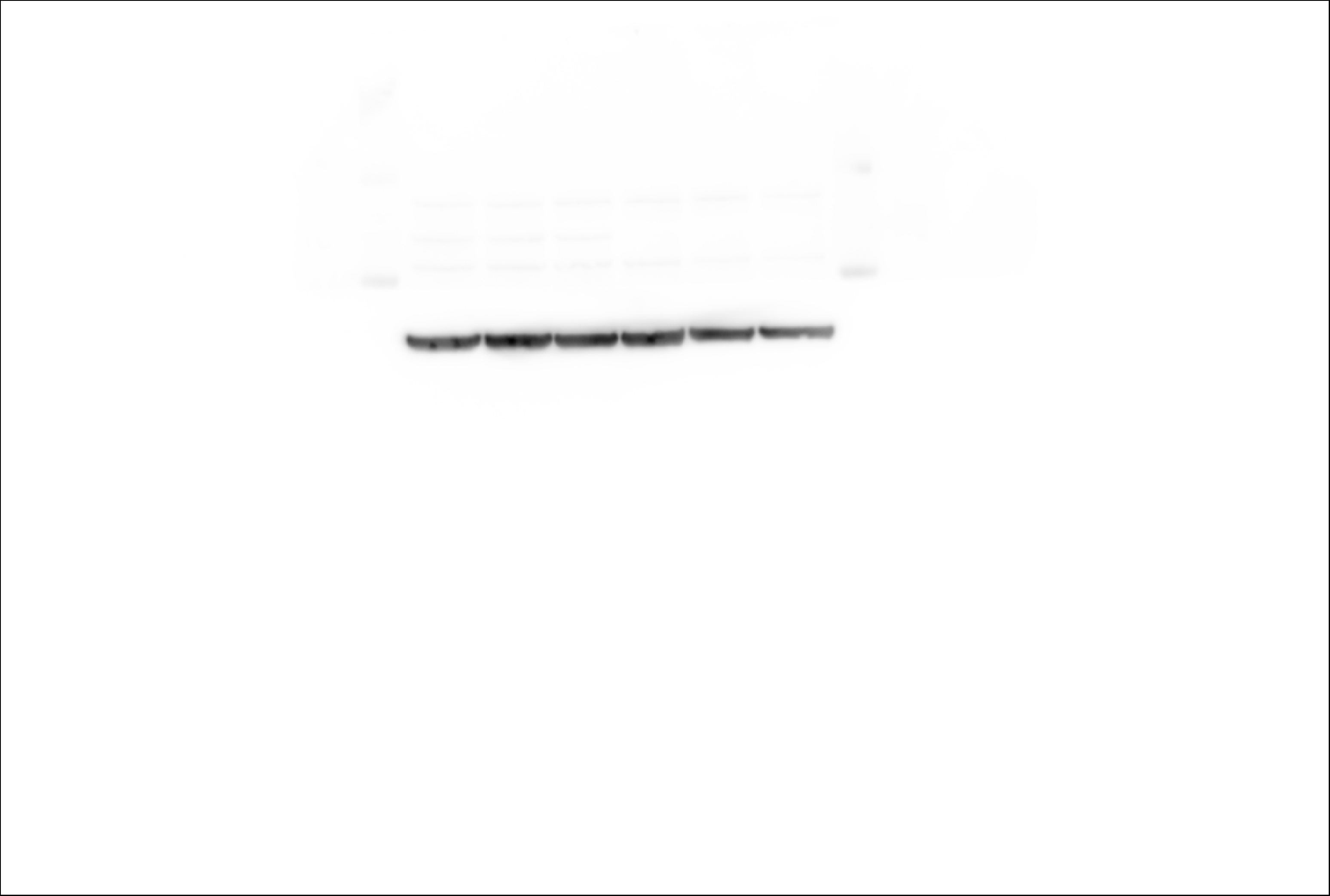

Supplement: Figure 1—source data 3. [file elife-80165-fig1-data3.zip › Figure1_B_C_D_source data/Figure_1D_raw_memberane2.tif]
