# Supplementary material for: The centrosomal protein 83 (CEP83) regulates human pluripotent stem cell differentiation toward the kidney lineage
Source: eLife. 2022 Oct 12;11:e80165. doi: 10.7554/eLife.80165 (PMC9629839; doi:10.7554/eLife.80165)
Supplement: Supplementary file 1. [file elife-80165-supp1.docx]

**Primers list used in the qPCR:**

| Gene | Forward primer sequence (5’ →3’) | Reverse primer sequence (5’ →3’) |
| --- | --- | --- |
| *CEP83* | AGACAGCAAACGAGTGGAAC | GGATCTGACTGTAGCCTGCA |
| *OSR1* | CCTTCCTTCAGGCAGTGAAC | CGGCACTTTGGAGAAAGAAG |
| *GAPDH* | AGCCACATCGCTCAGACAC | GCCCAATACGACCAAATCC |
| *OCT4/POU5F1* | AGCAAAACCCGGAGGAGT | CCACATCGGCCTGTGTATATC |
| *NANOG* | AAGGCCTCAGCACCTACCTA | ATTGGAAGGTTCCCAGTCGG |
| *SOX2* | CAAAAATGGCCATGCAGGTT | AGTTGGGATCGAACAAAAGCTATT |
| *HOXD11* | CAGCAGCGCAGTTGCC | CGGTCAGTGAGGTTGAGCAT |
| *EYA1* | AACAGCTCACCGTATCCAGC | TGTGCTGTACTCTGCTGTGG |
| *GATA3* | GCCCCTCATTAAGCCCAAG | TTGTGGTGGTCTGACAGTTCG |
| *HOXB7* | AAGCTCAGGAACTGACCGC | CCCTGTCTTGGCCGGTG |
| *PODXL* | CAACCCGGCCCAAGATAAGT | GGCAGGGAGCTTAGTGTGAA |
| *CUBN* | CTGCCGTCTTCCAGTCTCAG | ACAGCGGAACGAGCTTCTAA |
