# Supplementary material for: The centrosomal protein 83 (CEP83) regulates human pluripotent stem cell differentiation toward the kidney lineage
Source: eLife. 2022 Oct 12;11:e80165. doi: 10.7554/eLife.80165 (PMC9629839; doi:10.7554/eLife.80165)
Supplement: Supplementary file 2. [file elife-80165-supp2.docx]

**Primary antibodies used in IF staining:**

| Antibody | Dilution | Company |
| --- | --- | --- |
| Monoclonal Anti-Tubulin, acetylated antibody (T6793). mouse | IF: 1:2000 | Sigma-Aldrich, Saint Louis, MO, USA |
| anti-Cdh1 | IF: 1:200 | BD Bioscience, San Jose, CA, USA |
| Lotus tetragonolobus lectin (LTL) | IF: 1:200 | Vector lab, Burlingame, USA |
| Anti-NPHS1 | IF: 1:300 | R&D System, Minneapolis, MN, USA |
| Anti- CEP83 | IF: 1:200 | Sigma-Aldrich, Saint Louis, MO, USA |
| Cleaved Caspase-3 (Asp175). | IF: 1:200 | CellSignaling, Massachusetts, USA. |
